# Taking stories to the mountains

**DOI:** 10.3402/gha.v9.34405

**Published:** 2016-12-20

**Authors:** Mark Tomlinson

**Affiliations:** Department of Psychology, Stellenbosch University, Stellenbosch, South Africa, Email: markt@sun.ac.za

Children born in societies with a heavy social burden of HIV/AIDS, poor health and nutrition, and limited stimulation face additional challenges to flourishing before they turn six compared to children in societies that are not characterized by these environmental strains. Particularly, multiply-stressed caregivers, affected by HIV/AIDS and poverty, might have a limited capacity to provide care and stimulation to children, placing the latter at high risk for impaired cognitive and social/emotional development.

**Figure:**
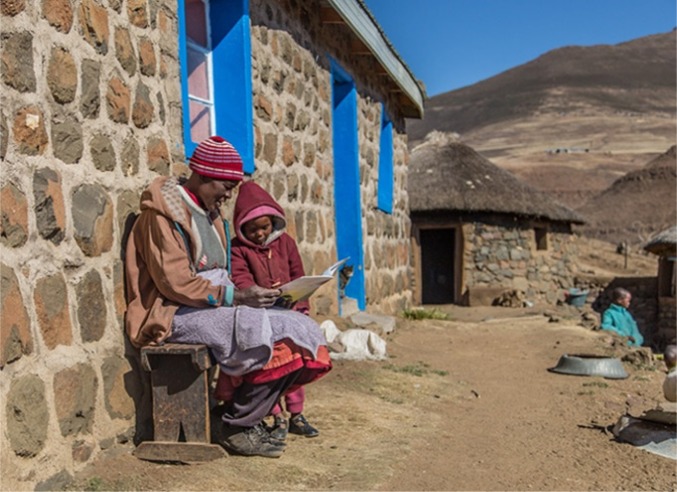
Chief Mabuoang Hlasa and a child reading a story in a book. Photographer: James Walsh, Sinamatella Productions, South Africa.

These risks can be minimized and possibly even be prevented by strengthening the family environment through the provision of high-quality programs for early childhood development, and – we believe – early book sharing.

Mphatlalatsane, which means ‘Early Morning Star’ in the local language, Sesotho, is a cluster randomized controlled trial to evaluate an integrated Early Childhood Care and Development intervention, including early cognitive stimulation, promotion of HIV testing and treatment support, and nutrition education in rural informal nursery care settings in the Mokhotlong district of Lesotho. The project is funded by PEPFAR-USAID.

The intervention is designed to increase early childhood stimulation, HIV testing and treatment, and child nutrition. As a part of this, caregivers of young children (aged 1–5 years) are receiving training in sensitive book-sharing skills – a strategy that stimulates the child cognitively and encourages caregiver–child engagement.

The research initiative is a collaborative project, led by Stellenbosch University, University College London, the University of Oxford, and the University of Reading, in collaboration with GROW, a local non-governmental organization, and the Ministry of Education and of Lesotho.

The author wishes to acknowledge the work of the following: Xanthe Hunt, Marguerite Marlow, Sarah Skeen, Peter Cooper, Lynne Murray, Lucie Cluver and Lorraine Sherr.

*Mark Tomlinson* Department of Psychology Stellenbosch University Stellenbosch, South Africa Email: markt@sun.ac.za

